# Antifungal Activity of Chitosan/Poly(Ethylene Oxide) Blend Electrospun Polymeric Fiber Mat Doped with Metallic Silver Nanoparticles

**DOI:** 10.3390/polym15183700

**Published:** 2023-09-08

**Authors:** Leire Murillo, Pedro J. Rivero, Xabier Sandúa, Gumer Pérez, José F. Palacio, Rafael J. Rodríguez

**Affiliations:** 1 Engineering Department, Public University of Navarre (UPNA), Campus Arrosadía S/N, 31006 Pamplona, Spain; murillo.127083@e.unavarra.es (L.M.); xabier.sandua@unavarra.es (X.S.); rafael.rodriguez@unavarra.es (R.J.R.); 2 Institute for Advanced Materials and Mathematics (INAMAT2), Public University of Navarre (UPNA), Campus Arrosadía S/N, 31006 Pamplona, Spain; 3 Genetics, Genomics and Microbiology Research Group, Institute for Multidisciplinary Research in Applied Biology (IMAB), Public University of Navarre (UPNA), 31006 Pamplona, Spain; gumer.perez@unavarra.es; 4 Centre of Advanced Surface Engineering, AIN, 31191 Cordovilla, Spain; jfpalacio@ain.es

**Keywords:** electrospinning, dip-coating, blend electrospun mat, chitosan, polyethylene oxide, LSPR, AgNPs, antifungal

## Abstract

In this work, the implementation of advanced functional coatings based on the combination of two compatible nanofabrication techniques such as electrospinning and dip-coating technology have been successfully obtained for the design of antifungal surfaces. In a first step, uniform and beadless electrospun nanofibers of both polyethylene oxide (PEO) and polyethylene (PEO)/chitosan (CS) blend samples have been obtained. In a second step, the dip-coating process has been gradually performed in order to ensure an adequate distribution of silver nanoparticles (AgNPs) within the electrospun polymeric matrix (PEO/CS/AgNPs) by using a chemical reduction synthetic process, denoted as in situ synthesis (ISS). Scanning electron microscopy (SEM) has been used to evaluate the surface morphology of the samples, showing an evolution in average fiber diameter from 157 ± 43 nm (PEO), 124 ± 36 nm (PEO/CS) and 330 ± 106 nm (PEO/CS/AgNPs). Atomic force microscopy (AFM) has been used to evaluate the roughness profile of the samples, indicating that the ISS process induced a smooth roughness surface because a change in the average roughness Ra from 84.5 nm (PEO/CS) up to 38.9 nm (PEO/CS/AgNPs) was observed. The presence of AgNPs within the electrospun fiber mat has been corroborated by UV-Vis spectroscopy thanks to their characteristic optical properties (orange film coloration) associated to the Localized Surface Plasmon Resonance (LSPR) phenomenon by showing an intense absorption band in the visible region at 436 nm. Energy dispersive X-ray (EDX) profile also indicates the existence of a peak located at 3 keV associated to silver. In addition, after doping the electrospun nanofibers with AgNPs, an important change in the wettability with an intrinsic hydrophobic behavior was observed by showing an evolution in the water contact angle value from 23.4° ± 1.3 (PEO/CS) up to 97.7° ± 5.3 (PEO/CS/AgNPs). The evaluation of the antifungal activity of the nanofibrous mats against *Pleurotus ostreatus* clearly indicates that the presence of AgNPs in the outer surface of the nanofibers produced an important enhancement in the inhibition zone during mycelium growth as well as a better antifungal efficacy after a longer exposure time. Finally, these fabricated electrospun nanofibrous membranes can offer a wide range of potential uses in fields as diverse as biomedicine (antimicrobial against human or plant pathogen fungi) or even in the design of innovative packaging materials for food preservation.

## 1. Introduction

Among all the fabrication techniques, the electrospinning process is being shown great interest in the scientific community because it presents several important advantages such as uniform pore diameter, ease of fabrication, low cost, great flexibility in surface functionality and high degree of reproducibility [1,2,3,4]. The fundamental of this technique is based on the application of a high voltage between a syringe filled with a polymeric solution with a desired viscosity and a collector mounted at a fixed distance from the needle/syringe setup [5,6,7,8]. The diameter of the fibers can be reduced from micro- to nanometer scale, enabling a large increase in the surface area-to-volume ratio and, as a result, several properties can be directly improved for many industrial (photocatalysis, anticorrosion and anti-icing) [9,10,11,12] or biomedical applications (tissue engineering, drug delivery and wound healing dressing) [13,14,15,16]. According to this last field, the use of biopolymers such as chitosan has received increasing interest in the medical sector due to its natural origin (abundant in nature) combined with exceptional properties in terms of biodegradability, biocompatibility and being a good inhibitor against the growth of a wide variety of yeasts, fungi and bacteria, diminishing infection of pathogens [17,18,19]. However, the resultant electrospinability of chitosan is very difficult because of the cationic nature of the solution, its rigid chemical structure and interaction of inter- and intra-chain hydrogen bonding [20]. One of the most successful strategies for enhancing the electrospinability of the chitosan is blending with a second natural polymer (i.e., hyaluronic acid, gelatin, zein and silk fibroin) [21,22,23,24] or synthetic polymer (i.e., polyvinyl alcohol- PVA, polyethylene oxide-PEO and polyacrylamide-PAM) [25,26,27,28,29] in order to form a polymeric blend with better physico-chemical and mechanical properties in comparison with pure chitosan [30]. In this sense, the use of PEO with its intrinsic characteristics (hydrophilic, biocompatible, non-cytotoxic and biodegradable) is a good candidate in the biomedical field [31], enabling the formation of uniform fibers with additional functionalities due to the specific interactions between amino groups of chitosan and ether groups of PEO [27,32]. More specifically, the formation of hydrogen bonds renders to chitosan a better electrospinability with an increase in the solution chain entanglements [33]. 

The incorporation of AgNPs into the electrospun fiber mats is a very interesting approach for the design of inorganic–organic hybrid materials [34] that can provide a synergistic antibacterial effect achieved by combining both AgNPs and chitosan [35]. The antimicrobial properties of chitosan are enhanced due to the presence of polymer chain’s positive charges and free amino groups [36], and it has been demonstrated that protonated amine groups of the chemical structure of the chitosan can bind negatively charged bacteria, breaking their reproduction [37]. These interactions lead to modified membrane permeability, interruption of the microbial membrane and subsequent leakage of proteins and other intracellular constituents that cause cell death [38,39,40]. In addition, thanks to the cationic-polyelectrolyte character and gel-forming ability related to the chitosan, this effect can be used as an effective binder of metal ions [31], the protonated amine and hydroxide sites being the main reactive sites for interactions with metal ions, whereas the use of chemical reduction methods is the most common synthetic route for the preparation of AgNPs into thin-films [41]. It is well known that AgNPs possess an inherent antibacterial activity against a broad spectrum of pathogenic microorganisms [42,43,44] on the benefit of their intrinsic optical colorimetric properties [45] associated to the Localized Surface Plasmon Resonance (LSPR) phenomenon, making possible the synthesis of nanoparticles with a desired shape, size and morphology [46]. Compared to bulk materials, the size, surface area and morphology of nanoparticles endow them with unique physical and chemical properties [47]. The experimental results also suggest that AgNPs can be used as effective growth inhibitors in various microorganisms, making them applicable to diverse medical devices and antimicrobial control systems [48,49]. There can be found in the bibliography more than one mechanism related to the antimicrobial activity of silver at the nanoscale [50,51,52,53]. 

In terms of antibacterial efficacy, a big number of publications are focused on the entrapment of the AgNPs into blended electrospun PEO/CS nanofibrous membranes in an only one-step fabrication process, showing promising results against Gram-positive bacteria (*Staphylococcus aureus* and *Propionibacterium acnes*) and Gram-negative bacteria (*Escherichia coli* and *Pseudonomas aeruginosa*) [54,55,56,57,58,59]. However, very few research publications have been found to be active against both bacteria and fungi by using this methodology of embedding AgNPs into blended electrospun fibers with the presence of chitosan [60,61]. The antimicrobial properties of the blended electrospun fiber mats with embedded AgNPs have been tested against a common skin pathogenic fungus such as *Candida albicans* by using the agar diffusion method. 

The implementation of hybrid inorganic–organic electrospun fiber mat against other fungi is of great interest. One of them is *Pleurotus ostreatus*, an edible filamentous basidiomycete with chitin and lignin degrader with important biotechnological properties and resources [62]. Their dikaryotic mycelia are the result of the fusion of two compatible monokaryons, which are formed in basidia after karyogamy (final step in the process of fusing two nuclei to form a unique diploid nucleus) and immediately undergo meiosis [63]. Previous works have shown that chitosan has great potential as an antifungal agent against ascomycetes fungus such as *Candida albicans*, *Fusarium solani* or *Aspergillus niger*, among others [64]. This compound can be obtained from *Pleurotus* sp. Some chitosan resistance strains of *P. ostreatus* have shown an overexpression on chitin deacetylases enzymes, which catalyze the deacetylation of chitin, result in the formation of chitosan [65,66]. An interesting approach for increasing the antimicrobial activity of the chitosan against a wide variety of fungi strains can be derived for a higher amount and more homogeneous distribution of the synthesized AgNPs on the outer surface of the blended polymeric electrospun matrix. One method that can provide this possibility is the in situ *synthesis* (denoted as ISS) process [67] where a polymeric matrix is firstly doped with silver ions (Ag^+^) and a further reduction step is performed in order to obtain AgNPs perfectly distributed along the thin-film surface [68]. The implementation nanofibers doped with AgNPs can be used in the design of active packaging to increase the shelf life of food or even to exert an inhibitory effect on fungal growth [69,70,71].

The novelty of this research work is the use of two different nanofabrication techniques such as electrospinning and dip-coating technology, which are totally compatible, enabling the development of highly effective antifungal surfaces, thanks to the combination of chitosan and AgNPs, respectively. For the fabrication of the functionalized thin films, a dual step is performed. Firstly, a PEO/CS blend electrospun nanofiber mat has been obtained and, secondly, the implementation of a chemical synthetic route (ISS process) is performed onto the electrospun mat with the aim of obtaining AgNPs, which are totally distributed in the outer surface. The resultant surface morphology (fiber diameter and roughness) of the electrospun nanofibers have been determined by SEM and AFM, whereas both UV-Vis spectra and EDX profile have been used for corroborating the presence of AgNPs within the polymeric fiber mat. Finally, notorious differences have been observed in terms of wettability and antifungal efficacy between blend nanofibers with and without AgNPs. To the best of our knowledge, this is the first time that the combination of both electrospinning and dip-coating techniques is presented in the bibliography for the design of functionalized surfaces against the fungus strain of *Pleurotus ostreatus*.

## 2. Materials and Methods

### 2.1. Materials

Chitosan (CS, medium molecular weight, ≥75% deacetylation degree), polyethylene oxide (PEO, 400 kDa), silver nitrate (AgNO_3_, ≥99%), borane dimethylamine complex (DMAB, 97%), glacial acetic acid (≥99%) and deionized water (DW) were purchased from Sigma-Aldrich (St. Louis, MI, USA) and used without any further purification.

### 2.2. Thin-Film Fabrication Process

The fabrication of the functionalized thin films was divided into two main steps. Firstly, electrospinning was used for the preparation of PEO/CS blend electrospun nanofibers. Secondly, the dip-coating technology was performed on the previously electrospun fiber mat in order to obtain AgNPs by a chemical reduction synthetic process (ISS). 

#### 2.2.1. Preparation of PEO/CS Blend Structure

The resultant PEO/CS blend solution was fabricated by mixing CS-acetic acid and PEO aqueous solution in a 1:1 (*v*/*v*) ratio. Initially, 5 wt% PEO solution dissolved in ultrapure water was prepared and, then, 4 wt% CS solution was prepared by using 90% acetic acid as solvent. Both solutions were left under magnetic agitation in a sealed container at room temperature during 24 h, being mixed at a 1:1 (*v*/*v*) ratio. Finally, the blend solution was left at room condition under magnetic agitation during 24 h, respectively, previous to perform the electrospinning process. 

#### 2.2.2. Electrospinning of PEO/CS Blend 

Electrospinning was performed using the “Electrospinning Professional Machine” from Doxa Microfluidics (Doxa Microfluidics, Malaga, Spain). The PEO/CS blend solution was introduced into a 5 mL syringe (BD Plastic, Franklin Lakes, NJ, USA) and pushed through a Teflon tube to a metal needle (inner diameter of 0.6 mm). The optimized experimental parameters were a flow rate of 1.3 mL/h, a distance of 20 cm from tip to collector and a specific voltage of 26 kV between the metal needle and the collector (V+ = 23 kV, V− = 3 kV), respectively. All the experiments were performed at room condition, showing a temperature of 23.7 °C and 32.45% of relative humidity (RH). Finally, all the coatings were exposed for a period of time of 90 min in the same experimental conditions. 

#### 2.2.3. In Situ Synthesis of Silver Nanoparticles (AgNPs)

**Figure 1:**
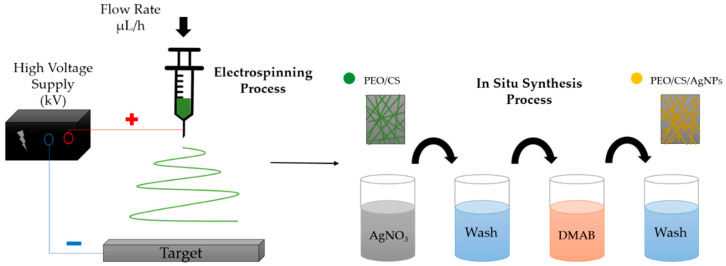
Schematic representation of the thin-film fabrication method used, separated into two steps: firstly, the production of PEO/CS electrospun nanofibers and, secondly, ISS process of AgNPs by using dip-coating technique onto the electrospun nanofiber mat.

In order to obtain the AgNPs, AgNO_3_ was used as the loading metallic agent (Ag^+^) and DMAB wasused as the reducing agent, which are necessary to perform the ISS process by using the dip-coating technique. The whole process is divided into the following steps. Firstly, the previously fabricated membrane was immersed in 0.01 M AgNO_3_ solution for 10 min, obtaining a membrane loaded with silver ions (Ag^+^). Then, this membrane was immersed in 0.1 M DMAB solution for 10 min, enabling the chemical reduction from silver ions (Ag^+^) to metallic silver (Ag^0^) nanoparticles. Between each loading and reduction step, the samples were immersed in ultrapure water (denoted as rinsing step). This same process was repeated to obtain a greater amount of AgNPs distributed in the polymeric electrospun membrane [66]. The dip-coating process was carried out by using ND-R Rotatory Dip-Coater (Nadetech Innovations, Navarra, Spain) [67]. Finally, a schematic representation of the whole procedure for the fabrication of the thin films is shown in Figure 1.

### 2.3. Characterization Techniques 

The morphology of the nanofibers was observed using a field-emission scanning electron microscope (FESEM, Hitachi S4800, Tokyo, Japan) and an atomic force microscope (AFM, Veeco Innova AFM, Veeco Instruments, Plainview, NY, USA). UV-Vis analysis was performed to confirm the formation of silver nanoparticles in the electrospun matrix. For this purpose, a Jasco V-730 (Agilent, Santa Clara, CA, USA) spectrophotometer was used. The analysis was carried out in a wavelength range of 350–900 nm. In addition, a thermogravimetric analysis (TGA) is performed by the TGA/DSC 3+ (METTLER-TOLEDO) in order to determine the amount of AgNPs in the electrospun samples. Three different process conditions can be distinguished during the thermal process. Firstly, it was measured in the temperature range from 35 °C to 775 °C with a nitrogen flow rate of 50 mL/min and a heating rate of 10 °C/min, respectively. Secondly, in the range from 775 °C to 800 °C, the resultant heating rate was decreased up to 5 °C/min, maintaining the same nitrogen flow rate. Thirdly, from 800 °C to 950 °C, an air flow rate of 50 mL/min was employed in order to eliminate the pyrolysis residues of the blend structure, maintaining the same heating rate. The chemical bonding and the presence of specific functional groups were analyzed by Fourier transform infrared spectroscopy using ATR mode (FTIR-ATR). The FTIR spectra were obtained by a Perkin Elmer Frontier spectrophotometer (Waltham, MA, USA) in the range of 650–4000 cm^−1^. Finally, the wettability of the surface was studied by measuring the contact angle with an optical tensiometer (CAM 100 KSV Instruments, Burlington, VT, USA). The water contact angle values (WCA) were calculated as an average of three measurements performed at different locations in each sample. 

### 2.4. Antifungal Test against “Pleurotus Ostreatus”

**Figure 2:**
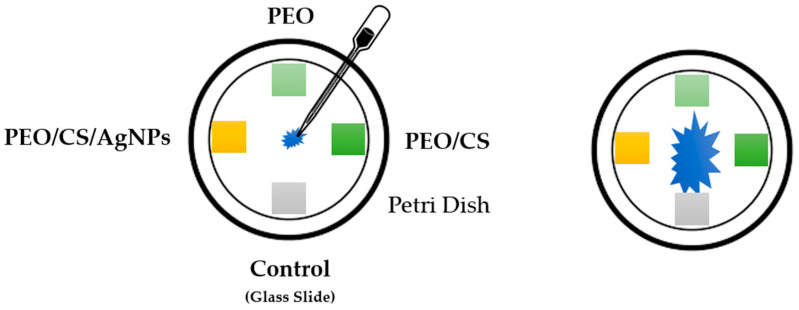
A schematic representation of the antifungal essay against the strain of *Pleurotus ostreatus* performed in this work.

The *Pleurotus ostreatus dikaryotic* strain dkN001 (Spanish Type Culture Collection accession CECT20600) was used in this work. This strain was cultured on square Petri dishes 120 × 120 mm containing Malt Extract Solid Medium (MESM: malt extract, 20 g/L; and bacteriologic agar, 15 g/L). Three glass slides were coated with PEO, PEO plus chitosan polymer (PEO/CS), and AgNPs plus PEO/CS sample (PEO/CS/AgNPs), whereas an uncoated glass slide was used as a control reference substrate, respectively. A piece of fungus mycelium was placed in the center of the Petri dishes and the three coated electrospun samples and the uncoated slide were placed at the end of the four axes of the Petri dish (see scheme of the Figure 2). Fungus was incubated in darkness conditions at 24 °C and the growth (apical form) was performed until the mycelium colonized the square Petri dish. 

The growth-inhibiting rate was calculated by the formula:

$$I=\frac{C-T}{C}\times 100\%$$

where “*I*” is the growth-inhibiting rate (%), while “*C*” and “*T*” represent the average colonization slides of the reference control (no electrospun fiber mat deposited) as well as the experimental electrospun coated glass slides (PEO, PEO/CS and PEO/CS/AgNPs samples), respectively. Data obtained were analyzed with IBM SPSS statistics 27.0 software (IBM Corp. Released 2020. IBM SPSS Statistics, Version 27.0. Armonk, New York: IBM Corp) using the Scheffe post hoc statistic to compare all possible simple and complex pairs of means between the different electrospun coated glass slides and the reference control slide. Finally, all the experiments have been evaluated in triplicate. 

## 3. Results and Discussion

**Figure 3:**
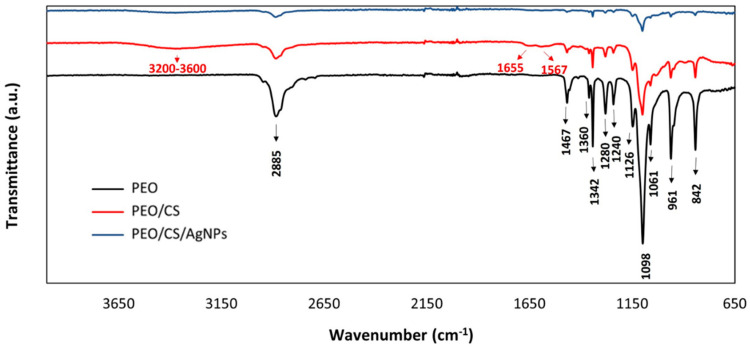
FTIR spectra of the electrospun samples: PEO (black plot), PEO/CS (red plot) and PEO/CS/AgNPs (blue plot), respectively.

The FTIR spectra of PEO, PEO-CS and PEO-CS+AgNPs films are presented in Figure 3. Additionally, the characteristic peaks of the spectra are marked. Those in black correspond to functional groups of PEO and those marked in red correspond to functional groups of chitosan, respectively. It should be noted that the red color spectrum contains both compounds (PEO and CS) so that, in addition to the peaks marked in red, there are also the peaks marked in black. The same is true for the blue spectrum. PEO shows absorption peaks at 2885 cm^−1^ (-CH_2_ stretching vibrations), at 1467 cm^−1^, 1360 cm^−1^, 1342 cm^−1^, 1280 cm^−1^, 1240 cm^−1^ and 842 cm^−1^ (all corresponding to CH bending vibrations) and, finally, at 1126 cm^−1^, 1098 cm^−1^, 1061 cm^−1^ and 961 cm^−1^ (C-O-C stretching vibrations) [72]. The characteristic absorption peaks of chitosan appear in the 3600–3200 cm^−1^ region, where a slight valley is observed (O-H and N-H stretching), at 1655 cm^−1^ (-C=O stretching) and at 1567 cm^−1^ (amide -NH_2_ bond) [73]. These last two peaks are conditioned by the degree of deacetylation of the chitosan, since the higher the degree, the greater the amount of glucosamine units and, therefore, the greater the presence of amino groups. Finally, no significant differences are observed between the FTIR spectra of the PEO/CS and the PEO/CS doped with AgNPs films.

**Figure 4:**
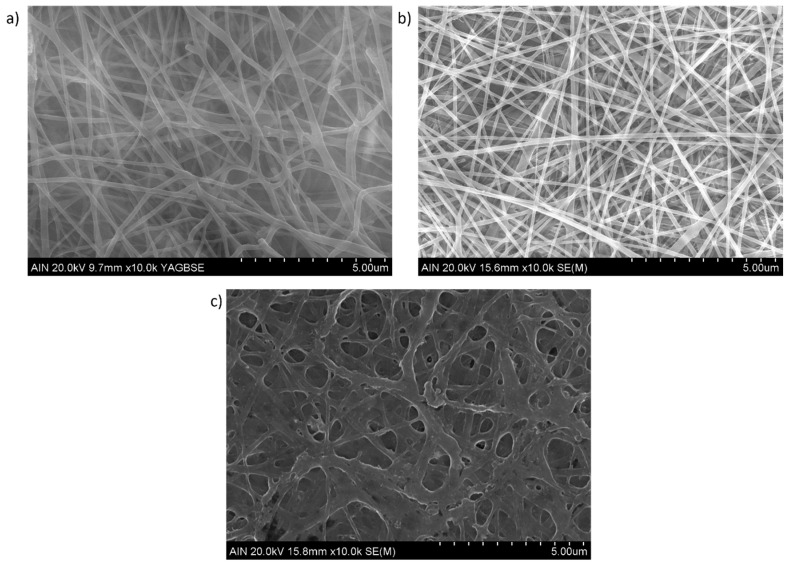
SEM images (scale bar 5 μm) of the blended electrospun fiber mats for PEO sample (**a**); PEO/CS sample (**b**) and PEO/CS/AgNPs sample (**c**), respectively.

**Figure 5:**
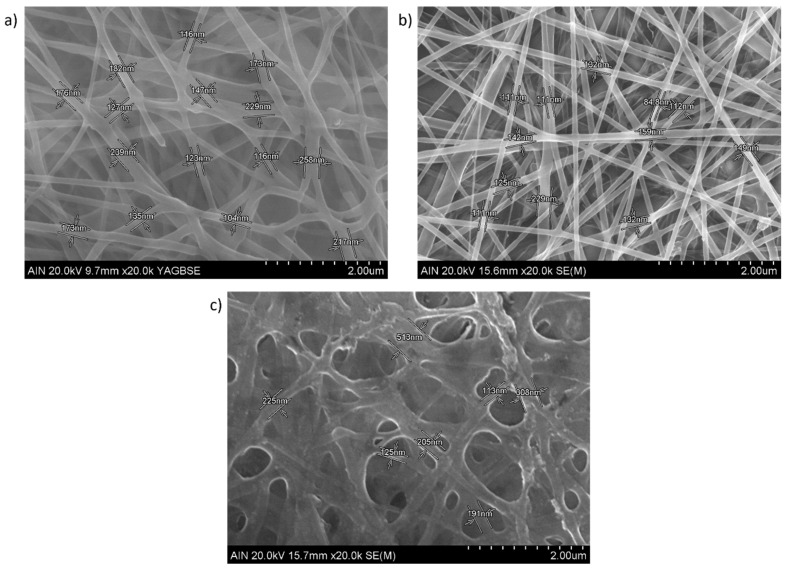
SEM images (scale bar 2 μm) of the blended electrospun fiber mats for PEO sample (**a**); PEO/CS sample (**b**) and PEO/CS/AgNPs sample (**c**) with the corresponding measurement of the fiber diameter.

**Figure 6:**
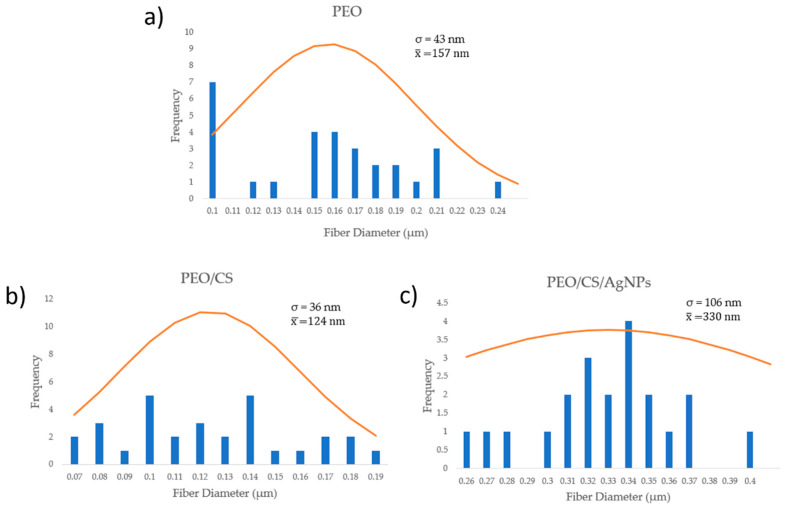
Nanofiber statistical diameter distribution of the electrospun mats for PEO sample (**a**), PEO/CS sample (**b**) and PEO/CS/AgNPs sample (**c**), respectively.

The surface morphology of the electrospun nanofibrous fiber mats is presented in Figure 4 (scale bar 5 μm) by using SEM micrographs. As has been previously mentioned, neat chitosan shows a very poor electrospinability and only beads or droplets are formed. It has been demonstrated that the addition of PEO can greatly facilitate the electrospinning process of chitosan in the blend at room temperature with the formation of beadless nanofibers [26]. In addition, the fiber diameter in the blended polymeric mat tends to decrease slightly when the CS amount is increased in the PEO/CS ratio [30]. In this work, uniform and defect-free nanofibers with diameters of 100–300 nm can be observed for PEO sample (Figure 4a) and PEO/CS sample (Figure 4b), showing a higher diameter for PEO/CS/AgNPs sample (Figure 4c). In order to clarify this result of the fiber diameter, Figure 5 presents SEM images (scale bar 2 μm) for all the samples of this study, showing different measurements of the nanofibers. The experimental results corroborate that an average fiber diameter of 157 ± 43 nm has been observed for PEO sample, whereas an average of 124 ± 36 nm has been obtained for PEO/CS sample. In the PEO/CS/AgNPs sample, the resultant fiber wall has become thicker, clearly indicating the integration of AgNPs within the nanofibers and, as a result, an increase in the fiber size has been clearly obtained, which is in concordance with the literature [56]. In this sense, the average in fiber diameter of 330 ± 106 nm has been observed for PEO/CS/AgNPs sample, showing a notable increase in comparison with the previous samples. According to this experimental result, Figure 6 shows the average corresponding to the diameter of the nanofibers as well as their corresponding distribution for each sample of this study [74]. The main reason for this phenomenon is associated to the hydrophilic behavior of both PEO and CS polymers, which show a high affinity to the aqueous media of the ISS process. As a result, a swelling behavior of the nanofibers can be appreciated with the corresponding increase in the fiber diameter. In addition, another relevant aspect is that the characteristic porosity associated to the electrospinning deposition process has been decreased because of this increase in the resultant fiber diameter after ISS process of the AgNPs. This result has been previously corroborated by performing the layer-by-layer deposition technique after the electrospinning process [12,75].

**Figure 7:**
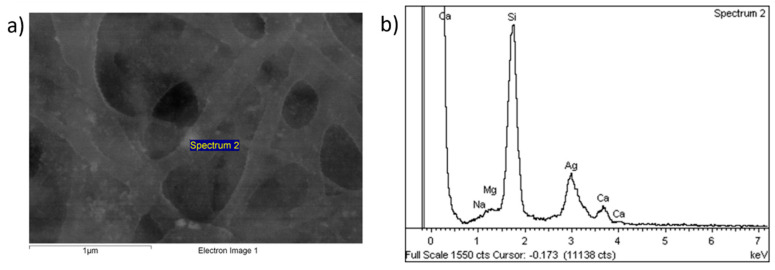
(**a**) SEM image of the blended electrospun fiber mat corresponding to the PEO/CS/AgNPs sample at scale bar of 1 μm; (**b**) EDX of the outer surface of the sample with the presence of Ag (bright spots).

In order to corroborate the presence of AgNPs in the outer surface of the blended electrospun fiber mat, energy-dispersive X-Ray (EDX) is presented in Figure 7, where the location of the peak related to Ag in 3 keV is clearly observed (see Figure 7b). However, the presence of other chemical elements (i.e., Si, Mg, Ca and Na) have also been observed in the EDX profile, which are inherent to the reference glass slides used for the fabrication of the electrospun nanofibrous coatings. The resultant SEM image also shows a random distribution of AgNPs in the complete outer surface with a certain degree of aggregation due to the formation of clusters combined with smaller-sized AgNPs (bright white spots) along the whole electrospun nanofibers. 

**Figure 8:**
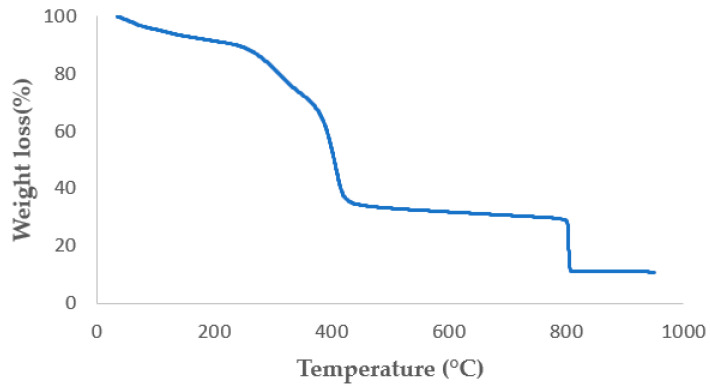
Thermogravimetric analytic curve for PEO/CS/AgNPs electrospun sample.

In order to determine with a high accuracy the resultant amount of AgNPs within PEO/CS/AgNPs sample, TGA analysis was carried out. In Figure 8, the percentage of weight loss of the sample in a temperature range from 35 °C up to 950 °C, respectively, is shown. The first weight loss corresponds to residual solvent (i.e., acetic acid) at 35 °C, which is still present in the sample. Then, the second weight loss is due to the degradation of the CS/PEO blend, which starts disintegrating at 210 °C, showing two slopes of disintegration, and typical TGA graphic shape when both CS and PEO polymers are blended [76,77]. After the degradation of the blend structure in a nitrogen atmosphere, a black appearance residue appeared due to the pyrolysis of the CS/PEO compound. This residue of pyrolysis prevents us from obtaining the amount of silver that remains in the specimen. Thus, the heating rate velocity is reduced and air is introduced in the chamber. At 800 °C, air is introduced, and this fact leads to a total disintegration of the pyrolysis residues of the blend structure to CO_2_, respectively. In this TGA figure, weight loss can be clearly observed as soon as the air is introduced in the chamber. Nevertheless, the remaining silver is oxidized, obtaining Ag_2_O product. Finally, the amount of silver is calculated from the remaining weight of the sample, taking into account the amount of Ag presented in the Ag_2_O sample. According to Equation (1), the percentage of silver presented in the Ag_2_O is obtained. In this attempt, the initial and final weight of the specimen in the TGA are 8.197 mg and 0.901 mg, respectively. According to this, Equation (2) is used to calculate the overall amount of silver in the electrospun sample from the remaining Ag_2_O product, obtaining a final value of 10.23% in the PEO/CS/AgNPs sample.

(1) $$Ag\%=\frac{{MW}_{Ag}}{{MW}_{O}+{MW}_{Ag}}=\frac{2\cdot 107.868}{15.999+2\cdot 107.868}=93.1\%\;of\;Ag\;in\;the\;{Ag}_{2}O\;molecule$$

(2) $$0.931\cdot \frac{{Ag}_{2}O\;Weight}{Initial\;Weight}=0.931\cdot \frac{0.901}{8.197}=10.23\%\;of\;Ag\;in\;electrospun\;sample$$

**Figure 9:**
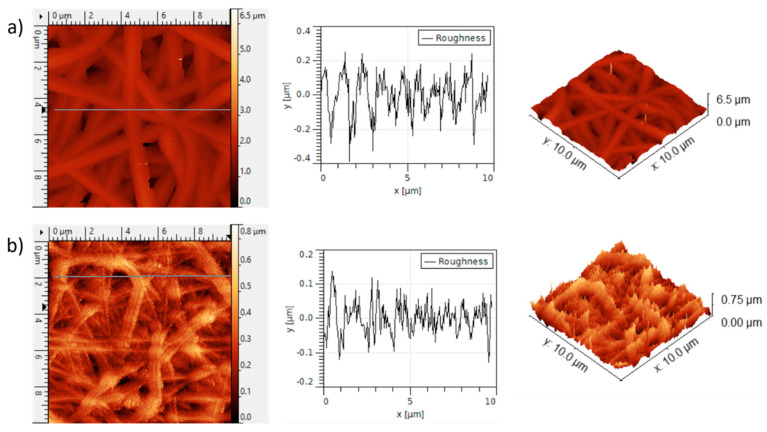
AFM images in 2D and 3D with their corresponding roughness profile for PEO/CS sample (**a**) and for PEO/CS/AgNPs sample (**b**), respectively.

AFM imaging has been performed in order to examine the morphology and to determine the surface roughness of the blended electrospun nanofibers. In Figure 9, two-dimensional (2D) and three-dimensional (3D) AFM images for PEO/CS sample (Figure 9a) and for PEO/CS/AgNPs sample (Figure 9b) can be observed. In this last sample, light spots are also observed corresponding to the AgNPs distributed over the blended electrospun fibers. In addition, the surface roughness was investigated with AFM by measuring the line profile of both samples, the average roughness R_a_ being 84.5 nm for PEO/CS sample and 38.9 nm for PEO/CS/AgNPs sample, respectively. This result corroborates that ISS process over the blended electrospun nanofiber mat clearly induces a smooth roughness surface, which has been corroborated by the AFM profile images. 

**Figure 10:**
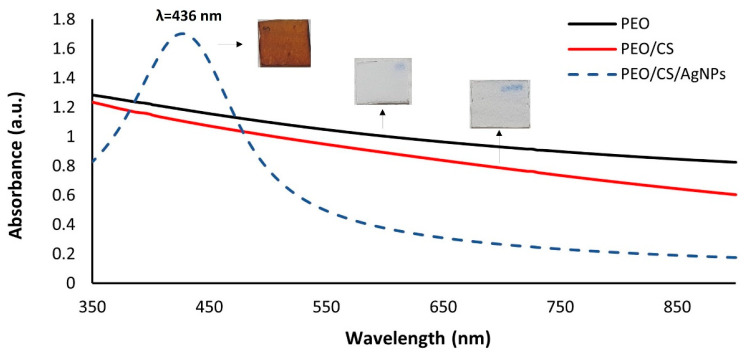
UV-Vis spectra of PEO sample (black plot), PEO/CS sample (red plot) and PEO/CS/AgNPs (dashed blue plot) with the presence of the LSPR absorption band centered at 436 nm inherent to the presence of AgNPs within the electrospun blended polymeric membrane.

Another way to corroborate the presence of the metallic AgNPs into the electrospun nanofiber mats is by using UV-Vis spectroscopy, and the UV-Vis spectra of all the samples of this study are shown in Figure 9. Significant differences can be extrapolated from these spectra data because, in one of them, an intense absorption band can be clearly observed in the visible region. First of all, an aspect indicative is that a dramatic color change from transparent (PEO or PEO/CS) to orange (PEO/CS/AgNPs) has been clearly observed after the two loading/reduction cycles of the ISS process (see inset of the Figure 10). This colored appearance is the result of the presence of a well-centered absorption band in the visible region inherent to the LSPR phenomenon, indicating the complete synthesis and incorporation of the AgNPs within the polymeric electrospun fiber mat [45,46]. 

Previous works have demonstrated that the amount of AgNPs is higher when the number of loading/reduction cycles is gradually increased, showing at the same time an increase in intensity of the LSPR absorption band, which is directly proportional to the amount of AgNPs trapped into the polymeric overlay [68,78]. The location of the LSPR absorption band is inherent to multiple factors such as shape, size, morphology, aggregation state, distribution and the resultant interparticle interactions of the nanoparticles, among others [79,80]. A previous work has demonstrated that LSPR absorption bands located at 600–700 nm are indicative of the presence of AgNPs with variable shape (i.e., rod, hexagonal and triangle), whereas LSPR absorption bands located at 400–500 nm indicate the presence of AgNPs with mostly spherical shape [81]. More specifically, the UV-Vis spectrum related to PEO/CS/AgNPs sample shows a maximum LSPR absorption band located at 436 nm, which is indicative of the synthesis of AgNPs with spherical shape with a variable size (polydispersity) and even associated to silver aggregates in the electrospun fiber mat, which has also been corroborated in the SEM image for EDX analysis [68]. 

**Figure 11:**
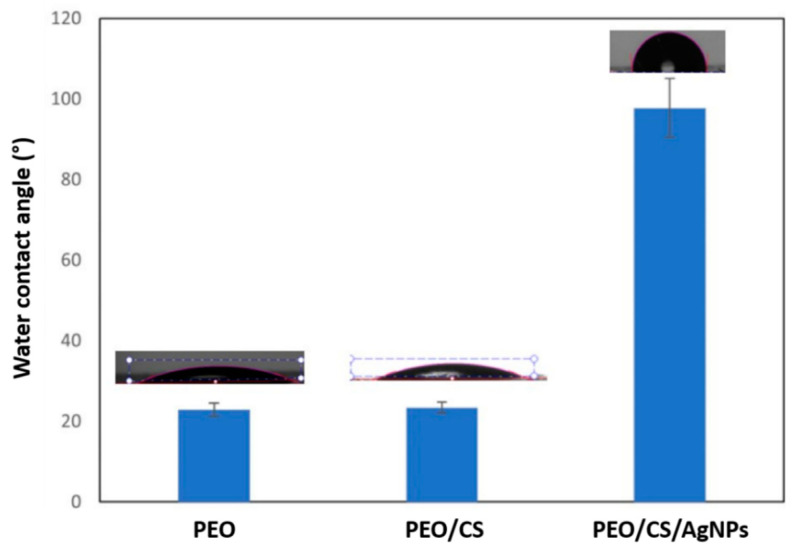
Water contact angle (WCA) values for all the samples of this study: PEO, PEO/CS and PEO/CS/AgNPs, showing an important difference in the resultant wettability from hydrophilic (PEO, PEO/CS) up to hydrophobic behavior (PEO/CS/AgNPs).

In Figure 11, the static water contact angle evolution of the electrospun samples of this work is presented. First, it is important to remark that, as a function of the resultant chemical composition as well as surface energy, the materials can vary their corresponding wettability degree [82]. The wettability of a material surface, i.e., the hydrophobicity or hydrophilicity, is often used as a primary descriptor of surface biocompatibility [83]. In this sense, high surface energy materials are relatively easy to wet out with a high degree of affinity (hydrophilic behavior), whereas low surface energy materials present a very little attraction to any molecule (hydrophobic behavior), without showing any chemical affinity. In the top of each column of Figure 11, the picture of a water droplet deposited onto the outer surface of each sample is shown, and a great difference in the resultant shape of this droplet can be clearly observed, which is inherent to its wettability. In this sense, a more hydrophilic surface is observed when the resultant water droplet is more dispersed onto the electrospun fiber mat, which corresponds to a smaller water contact angle value. In contrast, a more hydrophobic surface is obtained when the water droplet presents a more intrinsic spherical shape, which corresponds to a bigger water contact angle value. 

An initial interesting experimental result is that differences in the resultant wettability have not been observed for the PEO (22.9° ± 1.6) and PEO/CS (23.4° ± 1.3) samples, showing in both cases a hydrophilic nature, this being in concordance with the reported bibliography [84]. The main reason for this hydrophilic behavior can be associated to the presence of free polar groups in chitosan combined with the ether group of PEO, which show a great affinity to absorbing water molecules. The distribution of these functional groups on the outer surface of the electrospun nanofibers can reduce the interfacial tension and increase the affinity to water droplet, as the presence of this blend structure (PEO/CS) on polymeric matrices has been reported, even with hydrophobic nature [85]. However, performing the ISS of AgNPs onto the blend electrospun mat produced an important change in the resultant wettability by showing an important increase in the water contact angle. More specifically, the presence of the AgNPs confers a hydrophobic character to the PEO/CS blend electrospun mat, showing a water contact angle value of 97.7° ± 5.3, respectively. This experimental result of the PEO/CS/AgNPs can be associated to a dual effect. On one hand, previous research works have demonstrated that AgNPs have shown a hydrophobic behavior, indicating that surface properties can be tuned with AgNPs [86]. On the other hand, the increase in the resultant fiber diameter after doping with AgNPs by the ISS process (corroborated by SEM images) can contribute that the outer surface produces lower accessibility of the water droplets within the fiber mat with the corresponding increase in the water contact angle [87]. This increase in hydrophobicity is of interest for the design of a desired functionality in terms of anti-adherence activity [88] because it makes it difficult for microorganisms to colonize the surface [89]. 

**Figure 12:**
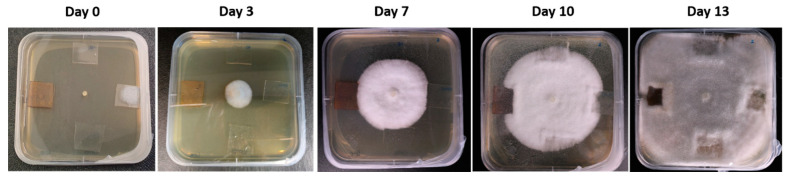
Evolution time related to the characteristic apical growth of the strain *Pleurotus ostreatus* from day 0 up to day 13 until a complete colonization of the Petri dish.

The analysis of the antifungal effect of the different samples on the growth of dkN001 strain of *P. ostreatus* was performed during a period of time of 13 days until fungus had completely colonized the Petri dishes (see Figure 12). First of all, the experimental results clearly indicate that, until the first week, the fungus showed an apical growth in all directions without touching the coated glass slides. However, after 10 days of exposure, apical hypha (filamentous structure of mycelium) established contact with all the coated glass slides, being more notorious after 13 days of exposure. 

**Figure 13:**
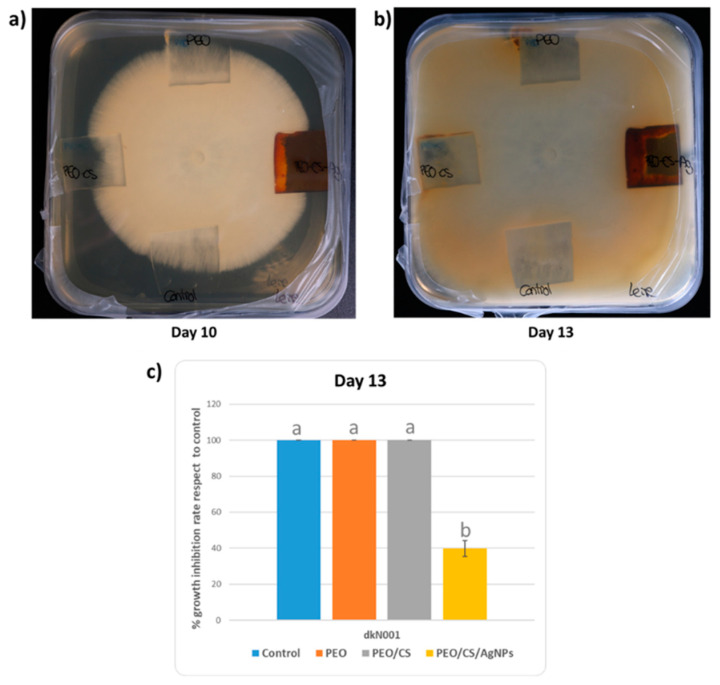
(**a**) Inhibition zone related to the PEO/CS sample (left axis) and PEO/CS/AgNPs sample (right axis) in comparison with PEO sample (superior axis) and control reference glass slide without coating (inferior axis) where the apical growth of the fungus strain after 10 days of exposure is clearly observed; (**b**) antifungal activity of the PEO/CS/AgNPs electrospun sample in comparison with PEO/CS sample, which is totally colonized after 13 days of exposure; (**c**) percentage inhibition of different electrospun samples in comparison to control after 13 days of exposure. Bars show the data average (n = 3) and SD (standard deviation). Lower case letters (a,b) indicate significant differences at *p*-value < 0.05 according to Scheffe’s test (see Materials and Methods).

In order to appreciate significant differences between all the samples of the study, in Figure 13, the evolution is presented in more detail corresponding to days 10 and 13, respectively. It can be observed that the mycelium has shown growth in a normal form continuing without perturbations in the axes where PEO sample and reference control glass slide (no electrospun fiber mat deposited) have been placed (day 10). However, an interesting result with important differences is that PEO/CS and PEO/CS/AgNPs samples showed an inhibition zone during mycelium growth in this period of time of 10 days (see Figure 13a). Once the whole Petri dishes were completely colonized after 13 days, only PEO/CS/AgNPs electrospun sample showed antifungal activity on the dkN001 strain of *P. ostreatus* (see Figure 13b,c), observing a mean decrease in the growth rate of 39.7% compared to reference control.

The antifungal effect of only chitosan polymer is well known [64], and it has been described that fungus such as *Cryptococcus* or *Pleurotus* can express chitin deacetylase genes during vegetative growth to provide integrity and proper rigidity to the cell wall and, in this way, cope with the effect of self-chitosan production [65,66]. In this work, the antifungal potency was clearly increased after doping the blended electrospun nanofibers with AgNPs in comparison with the other nanofiber formulation (PEO/CS) as per the zone of inhibition, which can be associated to their intrinsic hydrophobic nature and its antimicrobial behavior. According to the literature, AgNPs have emerged as effective antimicrobial agents by showing growth inhibition of different pathogen fungi, such as Aspergillus or *Candida*, among others. The antifungal mechanism related to the AgNPs can be associated to the production of reactive oxygen species (ROS) and free radicals, which can cause protein denaturation, nucleic acid and proton pump damage, lipid peroxidation, and cell wall damage. All these sequential steps enable an alteration in cell membrane permeability and, as a result, cell death is produced [90,91]. Thereby, it is also established that the stabilizing agents of AgNPs as well as the resultant surface charge can play an important role in the shaping of their fungicidal properties [92]. Finally, it is the first time that antifungal activity against *P. Ostreatus* has been demonstrated thanks to the combination of both AgNPs and chitosan by using this dual fabrication process of electrospinning and dip-coating techniques. 

## 4. Conclusions

In this work, a novel strategy is presented based on a dual-step fabrication process for the design of functional surfaces in terms of antifungal activity. In a first step, the electrospinning process was selected for the fabrication of PEO/CS blend structure, showing uniform and defect-free nanofibers with an average in fiber diameter of 124 ± 36 nm. In a second step, the dip-coating technology was used for the ISS of AgNPs onto the electrospun fiber mat, and the resultant PEO/CS/AgNPs has shown an increase in fiber diameter up to a value of 330 ± 106 nm, corroborated by SEM images. In addition, AFM analysis was used to evaluate the roughness profile of the samples, indicating that the ISS process induced a smooth roughness surface because a change in the average roughness Ra from 84.5 nm (PEO/CS) up to 38.9 nm (PEO/CS/AgNPs) was observed. The presence of the AgNPs was clearly observed by the change in the optical properties of the electrospun films from transparent (PEO/CS) to orange coloration (PEO/CS/AgNPs), respectively. This aspect is associated to the presence of an intense absorption band in the visible region, which is associated to the LSPR phenomenon. In addition, the EDX profile also corroborates the presence of silver (peak at 3 keV), whereas SEM images indicate that a mixture of smaller AgNPs combined with silver aggregates was obtained. After performing the ISS process, an amount of 10.23% of silver was present within the electrospun fibers (corroborated by TGA). These AgNPs in the outer surface of the nanofibrous mat confer an important change in the wettability with an intrinsic hydrophobic character, showing a variation in the water contact angle value from 23.4° ± 1.3 (PEO/CS) up to 97.7° ± 5.3 (PEO/CS/AgNPs), respectively. The antifungal activity was evaluated against *P. ostreatus* (showing a clearly apical growth) and the experimental results indicate that both samples PEO/CS and PEO/CS/AgNPs showed an inhibition zone after 10 days of exposure to the fungus, indicating the antifungal effect of the chitosan in comparison with both PEO and reference glass slide samples. Finally, after 13 days of exposure to the fungus, only PEO/CS/AgNPs sample clearly showed an antifungal efficacy because a mean decrease in the growth rate of 39.7% was observed in comparison with the rest of the samples. Finally, the great versatility of both nanofabrication techniques with a high degree of compatibility between them enables the design of advanced functional coatings in terms of antimicrobial surfaces for a wide variety of biomedical or industrial sectors.
